# Physiotherapy Rehabilitation for Compressive Myelopathy in a 12-Year-Old Girl: A Case Study

**DOI:** 10.7759/cureus.60785

**Published:** 2024-05-21

**Authors:** Nitika Chavan, H V Sharath, Tanvi S Varma, Anushri R Patil, Raghumahanti Raghuveer

**Affiliations:** 1 Department of Neurophysiotherapy, Ravi Nair Physiotherapy College, Datta Meghe Institute of Higher Education and Research, Wardha, IND; 2 Department of Paediatric Physiotherapy, Ravi Nair Physiotherapy College, Datta Meghe Institute of Higher Education and Research, Wardha, IND; 3 Department of Cardiovascular and Respiratory Physiotherapy, Ravi Nair Physiotherapy College, Datta Meghe Institute of Higher Education and Research, Wardha, IND

**Keywords:** quality-of-life, functional independence, physiotherapy rehabilitation, cranio-vertebral junction, cervical compression myelopathy

## Abstract

Myelopathy manifests in childhood and can be clinically categorized according to the site of injury (which may result in spinal syndrome) or the source (which may be nontraumatic or widely traumatic). Nontraumatic myelopathy can be caused by inflammatory, infectious, nutritional, metabolic, or ischemic factors. It may also be associated with systemic illnesses such as demyelinating disease, multiple sclerosis, or systemic lupus. Nonintentional harm is a significant factor to take into account in instances of traumatic myelopathy, which can frequently be linked to additional injuries. MRI and CT radiography help identify compressive myelopathy. We present the case of a 12-year-old girl who is right-hand dominant. She was in good health six months ago but recently began experiencing weakness in both of her lower limbs. An MRI of the brain revealed basilar invagination with stenosis of the foramen magnum, causing compressive myelopathy at the cranio-vertebral junction. The patient was operated on, followed by physiotherapy rehabilitation to improve functional independence and quality of life.

## Introduction

Any condition that results in neurologic impairments in the spinal cord is referred to as myelopathy. In causation, injuries are typically categorized as traumatic or nontraumatic [1]. The incidence of spinal injuries in children is between 2.7% and 9% of spinal injuries. Vascular, inflammatory, viral, or compressive disorders are the causes of nontraumatic myelopathies. When assessing myelopathy, some clinical symptoms can be identified depending on the spinal tract(s) implicated and the neuroanatomical location of the lesion [2]. Complete or transverse lesions can cause bilateral motor impairment, dysautonomia, and sensory loss. Upper cervical injuries result in spastic tetraplegia due to upper motor neuron involvement. In contrast, lower cervical injuries lead to areflexia, weakness, and fasciculation in the upper limbs as a result of lower motor neuron lesions. Additionally, sensory loss, incontinence, and spastic paraparesis occur below the lesion. Another example of isolated motor involvement is acute flaccid myelitis (AFM), caused by the poliovirus and characterized by flaccid paralysis, fasciculation, and areflexia. This suggests a major insult to lower motor neurons or anterior horn cells [3,4].

Different patterns of injury in children can be attributed to a higher head-to-body ratio, different trauma mechanisms, and the biomechanical properties of the young spine. Compared to adult instances, 60%-80% of injuries involve the spine (cervical), whereas only 5%-34% of the thoracolumbar spine are involved, with older children being more susceptible [5]. For children with compressive myelopathy, nonsurgical treatments exist. Analgesics, such as paracetamol, are used to relieve pain from inflammatory myelopathy. Nonsteroidal anti-inflammatory medications (NSAIDs) through corticosteroid injections are used to alleviate spinal cord edema. Spinal compression, which causes muscle weakness and bad posture, can be treated with physical therapy. Surgical intervention may be necessary in cases of moderate-to-severe myelopathy when nonsurgical treatments are ineffective or exacerbate the illness [6]. To remove any compressive material, such as tumors, bone fragments, or fluid accumulations, decompression surgery is required. The purpose of fusion operations is to preserve appropriate alignment and stabilize the spine.

Early detection and timely treatment are essential for improving the quality of life and minimizing long-term effects [7]. Improvements in strength and function may not be seen for months or years following presentation, but early physiotherapy is essential for preserving range of motion and should continue to maximize rehabilitation [8]. Exercise treatment and robotic technologies that help improve gait are two possible components of physiotherapy. Additionally, a large selection of customized orthotics and assistive devices based on age and degree of impairment are offered [9].

## Case presentation

**Figure 1:**
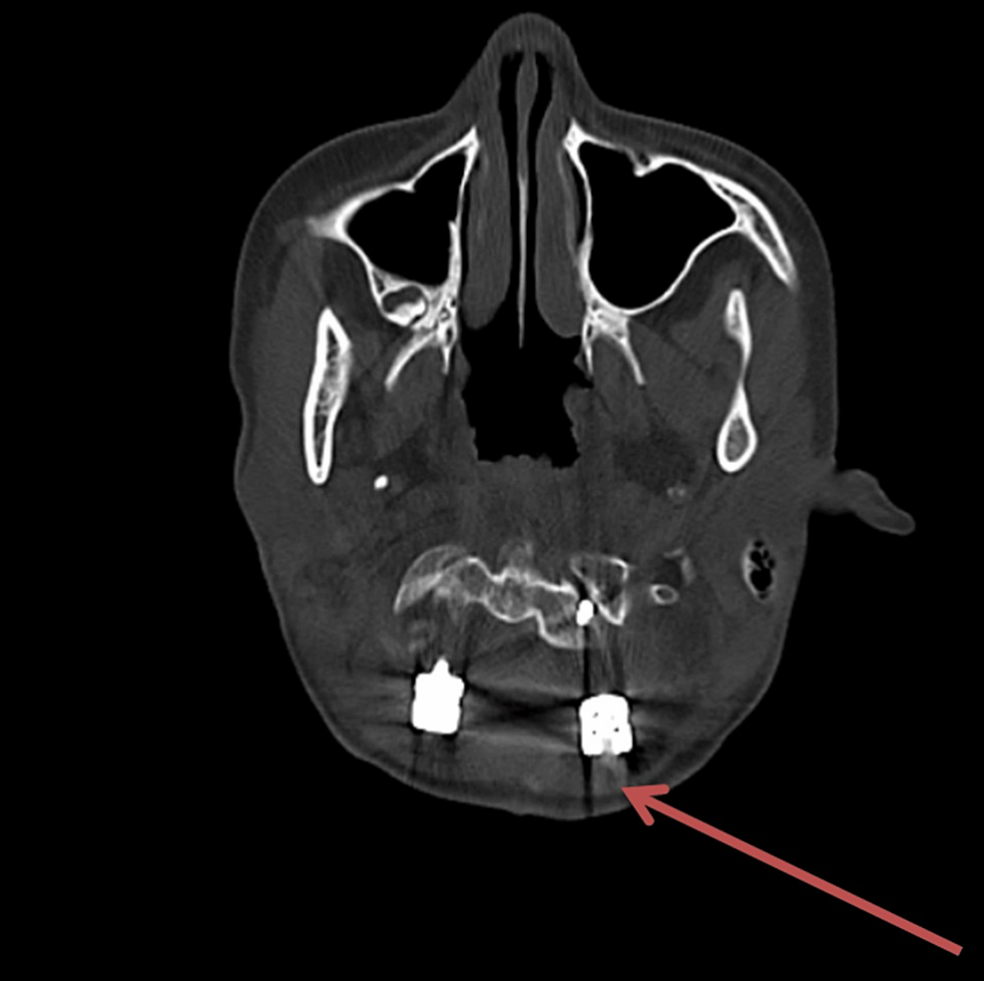
Brain CT scan showing the atlantooccipital assimilation with the C2-C3 fusion and basilar invagination compressing the cervico-medullary junction

A 12-year-old girl with right-hand dominance was in good health six months ago when she started complaining of weakness in her bilateral lower limbs. She started to have difficulty walking and wearing slippers. The weakness gradually progressed, and the child began to experience problems walking in straight lines (swaying movements). Two months ago, she reported experiencing neck movement difficulties and weakness in her right upper limb (extending to the distal hand), which progressed gradually. As a result, she began to struggle with her fine motor skills, such as holding a pen, buttoning and unbuttoning clothes, and coordinating movements. Subsequently, she was referred to a tertiary care hospital on January 8, 2024, and recommended to undergo an MRI scan. An MRI of the brain revealed basilar invagination with stenosis of the foramen magnum, causing compressive myelopathy at the cranio-vertebral junction. The patient was operated on January 10, 2024, for compressive myelopathy at the cranio-vertebral junction (Figure 1).

The childbirth documentation reveals that she was a female child weighing 2.8 kg, born to a primi mother at full-term gestation through a normal vaginal delivery (NVD). There is no record of any stay in the neonatal ICU. The child has achieved all developmental milestones appropriate for her age and has been fully immunized up to the present date. Currently, the child is experiencing difficulty in lifting objects with both her upper and lower extremities. Deep reflexes were assessed in pre-rehabilitation (Table 1).

**Table 1:** Reflexes +: diminished; ++: normal

Reflexes	Right (Pre-rehabilitation)	Left (Pre-rehabilitation)	Right (Post-rehabilitation)	Left (Post-rehabilitation)
Biceps reflex	++	++	++	++
Triceps reflex	++	++	++	++
Brachioradialis reflex	++	++	++	++
Supinator reflex	+	+	++	++
Patellar reflex	+	+	++	++
Achilles reflex	+	+	++	++

The superficial and cerebral reflexes were intact. Hyperreflexia was observed in both the extremities (upper and lower) below the site of the lesion. Manual muscle testing revealed paraesthesia or numbness in both hands (Table 2). Physiotherapy rehabilitation began on the first postoperative day following a thorough assessment that revealed difficulty performing fine motor movements due to weakness in the distal extremities. 

**Table 2:** Manual muscle testing

Joints	MMT Grade (Right) (Pre-rehabilitation)	MMT Grade (Left) (Pre-rehabilitation)	MMT Grade (Right) (Post-rehabilitation)	MMT Grade (Left) (Post-rehabilitation)
Shoulder flexors	2	2	4	4
Shoulder extensors	2	2	4	4
Elbow flexors	2	2	4	4
Elbow extensors	2	2	4	4
Wrist flexors	2	2	4	4
Wrist extensors	2	2	4	4
Hip flexors	1	1	3	4
Hip extensors	1	1	3	4
Knee flexors	1	1	3	4
Knee extensors	1	1	3	4
Ankle plantar flexor	1	1	3	4
Ankle dorsiflexors	1	1	4	4

The utilized outcome measures included the numerical pain rating, functional independence measure, dynamic gait index, Barthel index, and pediatric balance scale (Table 3).

**Table 3:** Outcome measures

Outcome Measures	Pre-rehabilitation (/n)	Post-rehabilitation (/n)
Numerical Pain Rating Scale (NPRS)	On activity: 7/10; on rest: 4/10	On activity: 4/10; on rest: 2/10
Functional independence measure (FIM)	50/126	110/126
Pediatric balance scale	11/56	46/56
Dynamic gait index (DGI)	05/24	22/24
Barthel index	35/100	90/100

The interpretation in Table 3 reveals that the child has shown improvement in all outcome measures after undergoing six weeks of physiotherapy rehabilitation.

Physiotherapy rehabilitation protocol

The patient underwent a six-week physical therapy rehabilitation program, receiving treatment for five days a week, with each session lasting 60 minutes (Table 4).

**Table 4:** Physiotherapy rehabilitation protocol

Problem	Goal	Intervention and Doses	Rationale
A complication may be more likely if the issue is not properly addressed	In order to minimize risks and complications	Describe the illness to the individual suffering from it, along with its advantages and safety measures	To increase the patient's autonomy and improve their overall well-being
Restrict the range of motion (ROM) in each of the lower and upper limbs	For ROM to get improved	Assisted mobility exercises with the help of a therapist (10 repetitions x 3 sets)	Executed because of diminished strength
Decreased range of motion in the cervical	To keep the cervical spine stable	Collar (cervical)	As the bones, muscles, and soft tissues in the neck recover, it supports the head's weight
The sufferer cannot stay upright	To enable the sufferer to stand on their own	A standing frame (for at least 15 minutes)	Facilitates adjusting to the antigravity posture
Affected posture	To keep proper alignment of the posture	A pillow placement in a proper manner	Boost self-esteem, relieves back pain
Challenges in coughing and swallowing	To facilitate the elimination of secretions	Exercises for expanding the thoracic region, i.e., thoracic expansion exercise (10 repetitions x 1 set)	Assists in enhancing chest flexibility and facilitates the elimination of fluids
Difficulty in breathing	To minimize the effort required for respiration	Deep breathing exercises (10 repetitions x 1 set)	To mitigate the frequency and intensity of pulmonary complications, such as pneumonia, atelectasis, and hypoxemia, it is crucial to implement measures that effectively reduce their occurrence and severity
Prolonged bed rest can lead to orthostatic hypotension	In order to alleviate the symptoms of lightheadedness or dizziness while seated, one can take certain measures	It is recommended to maintain a head-up tilt position. Additionally, it is advised to increase fluid intake and consider utilizing a tilt table	The tilt table offers a platform for enhancing the body's adaptation to gravity

## Discussion

The presented case study of physiotherapy rehabilitation for compressive myelopathy in a 12-year-old girl sheds light on several pertinent aspects of managing this challenging condition in pediatric patients. The primary objective of the study was to showcase the significance of physiotherapy rehabilitation in helping patients ultimately achieve their functional objectives. The successful rehabilitation outcomes observed in this case underscore the importance of early intervention and a multidisciplinary approach in managing compressive myelopathy in children. By promptly initiating physiotherapy interventions tailored to the individual needs and limitations of the patient, significant improvements in motor function, gait, and overall quality of life were achieved. This highlights the pivotal role of physiotherapists in optimizing functional outcomes and promoting long-term neurological recovery in pediatric patients with compressive myelopathy.

Moreover, the specific therapeutic modalities utilized in this case, such as targeted stretching exercises, gait training, and proprioceptive neuromuscular facilitation (PNF) techniques, demonstrate the effectiveness of evidence-based rehabilitation strategies in addressing the complex motor deficits associated with compressive myelopathy [10-13]. The comprehensive assessment and ongoing reassessment of the patient's progress allowed for the timely modification of treatment protocols, ensuring optimal therapeutic outcomes and minimizing the risk of secondary complications. Additionally, the collaborative efforts between the physiotherapy team, medical specialists, and the patient's family played a crucial role in achieving successful rehabilitation outcomes. By fostering open communication, providing education regarding the nature of the condition and the importance of adherence to the prescribed treatment plan, and offering emotional support, a holistic approach to care was established, thereby facilitating the patient's physical and psychological well-being throughout the rehabilitation process [14,15].

Currently, surgical techniques focusing on spinal support and decompression of the spinal cord are frequently utilized for treating cervical myelopathy [16]. For many decades, clinico-radiological and morphological examinations have documented cranio-vertebral anomalies. The natural history of patients with abnormalities at the cranio-vertebral junction is unclear. Clinical signs such as dysphagia, dysarthria, headaches, numbness, and restricted neck movements only appear later in life or following trauma [17]. An experienced physiotherapist prescribed numerous exercises for the patient in this report as part of a planned physical therapy recovery. Due to the limited movement in both lower extremities, the rehabilitation aimed to prevent bed sores and neurological complications. When a cervical collar is worn, the condition progresses more quickly. Patients with shorter durations of illness and minor cord atrophy in the neutral neck position are anticipated to experience improvement [18]. Early surgery has positive outcomes and can alleviate problems. It is imperative to provide care for patients experiencing mild symptoms.

Furthermore, the limitations and challenges encountered during the course of rehabilitation in this case warrant consideration. Factors such as the variability in individual patient responses to treatment, the potential for disease progression or recurrence, and the presence of comorbidities may influence the overall prognosis and necessitate ongoing monitoring and adjustment of the rehabilitation program. Future research endeavors should aim to address these gaps in knowledge through longitudinal studies with larger sample sizes and standardized outcome measures, thereby further elucidating the optimal management strategies for compressive myelopathy in pediatric populations [19,20].

## Conclusions

The conservative approach to compressive myelopathy includes pharmacologic interventions, neck immobilization, a proper exercise schedule, changes in diet, and physical therapy. To improve outcomes following surgery, the customized physical therapy regimen should be adhered to prior to any surgical procedure. A clear indication of the need for emergency surgery would be a total loss of strength in the arms and legs. This patient was treated surgically for compressive myelopathy at the cranio-vertebral junction. To achieve the best results, rehabilitation protocols should be thoroughly established as soon as possible. The most common treatments for this illness involve increasing muscle strength, improving the range of active and passive movement, and preventing further functional loss.

## References

[1] New PW, Lee BB, Cripps R, Vogel LC, Scheinberg A, Waugh MC (2019). Global mapping for the epidemiology of paediatric spinal cord damage: towards a living data repository. Spinal Cord.

[2] Dogan S, Safavi-Abbasi S, Theodore N (2007). Thoracolumbar and sacral spinal injuries in children and adolescents: a review of 89 cases. J Neurosurg.

[3] Basu S (2012). Spinal injuries in children. Front Neurol.

[4] Kotrashetti VA, Sonawane VB, Nair SR, Bainade K, Vatkar A, Gupta S (2020). Compressive myelopathy presenting with paraparesis in pediatric age. Ped Rev: Int J Ped Rev.

[5] Horovitz DD, Magalhães Tde S, Pena e Costa A, Carelli LE, Souza e Silva D, de Linhares e Riello AP, Llerena JC Jr (2011). Spinal cord compression in young children with type VI mucopolysaccharidosis. Mol Genet Metab.

[6] Hopkins SE (2017). Acute flaccid myelitis: etiologic challenges, diagnostic and management considerations. Curr Treat Options Neurol.

[7] Peev N, Komarov A, Osorio-Fonseca E, Zileli M (2020). Rehabilitation of spinal cord injury: WFNS Spine Committee recommendations. Neurospine.

[8] Stoner KE, Abode-Iyamah KO, Fredericks DC, Viljoen S, Howard MA, Grosland NM (2020). A comprehensive finite element model of surgical treatment for cervical myelopathy. Clin Biomech (Bristol, Avon).

[9] Singal K, Gupta PD, Gupta N (2019). Cranio-vertebral junction anomaly - presenting as cervical myelopathy. IJHHS.

[10] Hirayama K (2000). Juvenile muscular atrophy of distal upper extremity (Hirayama disease). Intern Med.

[11] Burile G, Jawade S, Seth N (2023). The scope of physiotherapy rehabilitation in compressive myelopathy managed by spinal fusion: a case report. Cureus.

[12] Srushti Sudhir C, Sharath HV (2023). A brief overview of recent pediatric physical therapy practices and their importance. Cureus.

[13] Bravar G, Luchesa Smith A, Siddiqui A, Lim M (2021). Acute myelopathy in childhood. Children (Basel).

[14] Garstang SV, Miller-Smith SA (2007). Autonomic nervous system dysfunction after spinal cord injury. Phys Med Rehabil Clin N Am.

[15] Absoud M, Greenberg BM, Lim M, Lotze T, Thomas T, Deiva K (2016). Pediatric transverse myelitis. Neurology.

[16] Karlsson AK (2006). Autonomic dysfunction in spinal cord injury: clinical presentation of symptoms and signs. Prog Brain Res.

[17] Fung GP, Chan KY (2003). Cervical myelopathy in an adolescent with Hallervorden-Spatz disease. Pediatr Neurol.

[18] Mondal A, Giri PP (2018). Cervical myelopathy in a child: a rare cause of hypoventilation syndrome presenting with type 2 respiratory failure. Indian J Crit Care Med.

[19] Butts R, Legaspi O, Nocera-Mekel A, Dunning J (2021). Physical therapy treatment of a pediatric patient with symptoms consistent with a spinal cord injury without radiographic abnormality: a retrospective case report. J Bodyw Mov Ther.

[20] Dho YS, Kim H, Wang KC (2018). Pediatric spinal epidural lymphoma presenting with compressive myelopathy: a distinct pattern of disease presentation. World Neurosurg.

